# Conceptual Models and Theories Applied to Nursing Education in Intercultural Contexts: State of the Art*

**DOI:** 10.17533/udea.iee.v41n2e14

**Published:** 2023-08-28

**Authors:** Adriana Lucia Valdéz Fernández

**Affiliations:** 1 Nurse, Ph.D. Professor, Universidad del Cauca, Popayán (Colombia). Email: adrianitalvf@unicauca.edu.co https://orcid.org/0000-0002-3314-3804 Universidad del Cauca Universidad del Cauca Popayán Colombia adrianitalvf@unicauca.edu.co

**Keywords:** cultural competency, education, nursing, models, nursing, nursing care, competencia cultural, educación en enfermería, modelos de enfermería, atención de enfermería, competência cultural, educação em enfermagem, modelos de enfermagem, cuidados de enfermagem

## Abstract

**Objective:**

To analyze academic production about nursing models and theories in intercultural contexts applied to the field of education.

**Methods:**

State-of-the-art study, which examined 50 articles from research.

**Results:**

Application of the cultural competence model was found as a trend at disciplinary level, and in at interdisciplinary level, critical pedagogy was used. Regarding the curriculum, it is observed that cultural competency is a subject that is taught, but it is not treated in transversal manner. The principal didactics was cultural immersion, which permits acquiring skills and aptitudes to care for diverse population. The evaluation in the educational act centered on characterizing the level of acquisition of cultural competency. The gaps indicate the difficulty of applying the models and theories in practice scenarios, while the recommendations focus on the importance of teacher training in cultural competency.

**Conclusion:**

Interculturality is approached as a borrowed theory that, from education, contributes to the nursing practice from training that vindicates situational knowledge and its articulation with ethics permits developing skills to relate with others who have their own views regarding health care.

## Resumen

**Objetivo:**

Analizar la producción académica sobre modelos y teorías de enfermería en contextos interculturales aplicados al campo de la educación.

**Métodos:**

Estado del arte donde se examinaron 50 artículos producto de investigación.

**Resultados:**

Se encontró como tendencia a nivel disciplinar la aplicación del modelo de competencia cultural y en cuanto a interdisciplinar se trabajó con la pedagogía crítica. Respecto al currículo, se observa que la competencia cultural es una temática que se imparte, pero no es tratada de forma trasversal. La principal didáctica fue la inmersión cultural, que permite la adquisición de habilidades y aptitudes para la atención con población diversa. La evaluación en el acto educativo se centró en caracterizar el nivel de adquisición de competencia cultural. Los vacíos señalan la dificultad de la aplicación de los modelos y teorías en los escenarios de la práctica, mientras que las recomendaciones se centran en la importancia de la formación docente en competencia cultural.

**Conclusión:**

La interculturalidad es abordada como una teoría prestada que, desde la educación, contribuye a la práctica enfermera desde una formación que reivindica los saberes situacionales y su articulación con la ética permite el desarrollo de habilidades para relacionarse con los otros que tienen visiones propias respecto al cuidado de la salud.

## Resumo

**Objetivo:**

Analisar a produção acadêmica sobre modelos e teorias de enfermagem em contextos interculturais aplicados ao campo da educação.

**Métodos:**

Estado da arte onde foram examinados 50 artigos de produtos de pesquisa.

**Resultados:**

A aplicação do modelo de competência cultural foi encontrada como uma tendência ao nível disciplinar e em termos de interdisciplinaridade, utilizou-se a pedagogia crítica. Em relação ao currículo, observa-se que a competência cultural é uma disciplina que se ensina, mas não é tratada de forma transversal. A principal didática foi a imersão cultural, que permite a aquisição de habilidades e aptidões para o atendimento a uma população diversificada. A avaliação no ato educativo centrou-se na caracterização do nível de aquisição da competência cultural. As lacunas apontam para a dificuldade de aplicação dos modelos e teorias em contextos de prática, enquanto as recomendações centram-se na importância da formação de professores em competência cultural.

**Conclusão:**

A interculturalidade é abordada como uma teoria emprestada que, a partir da educação, contribui para a prática de enfermagem a partir de um treinamento que reivindica o conhecimento situacional e sua articulação com a ética permite o desenvolvimento de habilidades para se relacionar com outras pessoas que têm suas próprias visões em relação à assistência à saúde.

## Introduction

Despite the importance of having epistemic and ontological support of care to train future nursing professionals, there is still scarce research from education on intercultural contexts.^1^ This indicates the need to inquire on what approaches have been carried out, bearing in mind that society continuously undergoes transformations that require the views and knowledge of others that contribute to more pertinent training^2^ and contextualized care. A situation evident in Latin America and Colombia, where these transformations are forced to cultural diversity, underscoring the need for the intercultural view, due to the forgetfulness of this situated knowledge that permit knowing how to live and coexist in a given setting.^3^ Thus, it is expected for education to serve as “counterweight to the anthropological and cosmological model imposed with the hegemony of the neoliberal spirit”.^3^

Likewise, in the education of nursing professionals, interculturality can promote a pedagogy that, in the words of Fornet-Betancourt, recovers the contextual knowledge of health care, rather than “disregarding the so-called traditional knowledge generated in and for the various life worlds”.^3^ Then, the need emerges to train nursing professionals competent to work with diverse populations ^4^ because interculturality goes beyond recognizing and accepting cultural diversity.^5^ Thus, from education, interculturality is defined as: “An attitude and intellectual, ethical, political, and social disposition regarding a relationship among culturally diverse people and social groups, where each one places themselves in continuous questioning and transforms in conditions of respect and dignity to construct other ways of thinking, being, doing, and coexisting”.^6^ This enables rethinking that, in health, interculturality implies “the explicit incorporation of the patient’s collective cultural burden in the relationship established with the health worker”.^7^ In nursing, weaving interculturality into the pedagogical act permits recognizing others as “human beings equal to them, but diverse in their thinking regarding health care, without, in this relationship, one of the actors locking themselves in their vision or one of them assimilating the gaze of the other and losing their identity, which would permit advancing toward a more reciprocal vision of care”.^5^

The philosophical visions of nursing care guide the training of the professional who must have an epistemic as of where,^8^ this sustenance can be given according to Barret^9^ from models and theories, both own and borrowed from other disciplines; The first, refer to the advances from the discipline that normally focus on the application of knowledge already established, while the second allude to development with other disciplines that permit advancing nursing knowledge from the practice or can perpetuate a hegemonic view of scientific knowledge. All these observations evidence the importance of selecting conceptualizations that support training professionals, bearing in mind the research and application experiences in the practice. Chrizostimo and Brandão^10^ reiterate that in the discipline, professional teaching presents a trend that ignores the context and social commitment, which results critical when considering that health systems demand more human aspects in care.^11^ Stemming from the aforementioned, a state-of-the-art was conducted, seeking to analyze the academic production on nursing models and theories in intercultural contexts applied to the field of education.

## Methods

**Figure 1:**
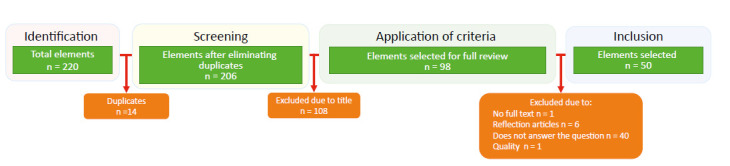
Flow diagram

This was a state-of-the-art qualitative research, which followed five phases:^12^ (i) *Contextualization*: corresponds to the definition of the criteria for the search for information, research limits, and types of resources to use. The search for the documents took place from June to December 2021 through the Descriptors in Health Sciences (DeCS): *Educación en enfermería*, *modelos conceptuales*, *competencia cultural* and their corresponding terms in English. Each of these was entered into the databases chosen: PUBMED, Web of Science, Scopus, CUIDEN, EBSCO, ERIC, LILACS, ProQuest, Redalyc, and university repositories. The inclusion criteria required the documents to be research results, available in English or Spanish, and no limit was established for date of publication, given that it was an emerging topic. The work excluded reflections, letters to the editor, essays, or studies not dealing with the topic of interest. At resource level, the Rayyan Systematic Review software was used to quickly read and select the articles and the Atlas.ti software version 22 for coding and analysis of the information. (ii) *Analysis:* permits classifying the documents regarding the methodological, epistemic, and pedagogical approach. This was done by loading onto the Rayyan software the studies obtained through the search. This software permitted reading the titles and abstracts, to select the research by eliminating duplicates and facilitated the application of exclusion criteria, forming a documentary *corpus* of 50 articles (Figure 1). These were described according to their characteristics and were categorized with respect to topic trends, by using Atlas.ti, where open codes were underlined.

(iii) *Interpretation by thematic nucleus*: broadens the study horizon relating the analysis units identified in the previous step, which transcends from a descriptive view to the creation of families that permit outlining useful hypotheses for the theoretical construction. (iv) *Global theoretical construction*: where the information extracted from the previous interpretation permits indicating the gaps, difficulties, trends, and achievements available currently in the field of study, for which the Atlas.ti software was used through the relations emerging from the families, thus, forming the core or selective category. And, (v) *Extension*: corresponds to the process of writing the article and submitting it to publication to disclose the results.

## Results

The characteristics of the articles included (Table 1) indicate predominance of qualitative research, a topic explored mostly since 2012, developed principally in the United States and with student population.

**Table 1:** Characteristics of the articles included

Parameter	Description of characteristics
Types of articles	*Quantitative* (15): Transversal (11), quasi-experimental (4)
	*Qualitative* (30): systematic literature review (8), phenomenology (5), case study (5), documentary analysis (2), integrative review (2), ethnography (2), content analysis (2), state of the art (1), narrative (1), community-based participative research (1), constant comparison method: theory of cultural contracts (1).
	*Mixed* (5).
Year of publication	2005 (1), 2007 (1), 2008 (3), 2009 (1), 2010 (2), 2011 (2), 2012 (5), 2013 (5), 2014 (2), 2015 (4), 2016 (4), 2017 (4), 2018(5), 2019 (6), 2020 (1), 2021 (4).
Country	The United States (23), Australia (4), Canada (3), Türkiye (3), Chile (2), Spain (2), Finland (2), Iran (2), Brazil (1), China (1), Colombia (1), South Korea (1), Slovenia (1), Mexico (1), Puerto Rico (1), Sweden (1), Venezuela (1).
Population approached	Students (18), documents (13), professors (6), nursing professionals (5), students and professors/university managers (3), university and faculty administrators (3), culturally diverse population (2).

The disciplines that support intercultural conceptual and theoretical models in the field of education, their trends and research gaps found in the review are summarized by the following: 

### Disciplines that support theories and conceptual models

It was identified in the training of nurses in intercultural contexts, theories and disciplinary and interdisciplinary models for nursing.^9^ Thus, the practice of this profession has been carried out from its own progress and at the same time it has been nourished by borrowed theories or interdisciplinary models that have been used in the practice, principally from education, psychology, and sociology, and to a lesser extent in philosophy, politics, anthropology, and ethics.

### Trends in conceptual and theoretical models

From the theoretical culture category, diverse open codes emerge and from the weave of its relations these are integrated to axial categories that concentrate nursing models and theories or for nursing. The following describe those with the greatest conceptual density throughout the studies reviewed.

### Own theories and disciplinary models

Theory of transcultural nursing. Also called the rising sun model, developed principally by Leininger, it is defined as a scientific and humanistic area that considers the differential individual characteristics of the population regarding the specifics of their cultural context, thereby, it is important to know the differences and similarities presented by cultures with respect to values of care, care practices, and of life;^13^^-^^16^ given that their knowledge will enable improving patient care, upon contemplating relevant information that permits managing effectively their health needs,^15^ recognizing the role and importance of culture on the health of individuals.^16^ This theory has been worked by other authors, like Williamson & Harrison^17^ and Cai^18^^,^^19^, the first consider that transcultural nursing is a competence of cultural care in holistic manner, which permits maintaining or reestablishing the health of individuals in an appropriate and beneficial way, the latter adds that skills can be developed to relate properly with other cultures at the moment of care, through encounters of two or more cultures.^20^

Cultural competency model. The studies mostly retake it from Campinha-Bacote, who defines it as the “skill of understanding the beliefs, values, behaviors, and customs to work effectively according to the client’s cultural context”.^21^ It is considered that the nursing professional requires a continuous process to acquire said skill of working with different cultures;^20^ consequently, the person is not, but becomes culturally competent.^15^^,^^22^ Thus, cultural competency, as a conscious process, seeks to adapt care so that it is consistent with the client’s culture.^23^ Hence, the person is the center of care, thereby, caregivers are required to incorporate in their behavior thoughts, actions, and ways to communicate with and from the other.^24^ This way, health professionals must make a constant effort to acquire and apply the cultural views of care in the relation established during the care process; this implies the development of five components: cultural awareness, cultural knowledge, cultural skill, cultural encounter and cultural desire.^20^ In addition, caregivers can provide what is denominated culturally competent care, understood as a sensitive and profound form of providing health, based on the needs of people’s ways of life so they have well-being.^20^ In order to achieve a holistic view that takes into account the individual’s needs considering their cultural differences.^25^

### Borrowed theories and interdisciplinary models

Theory of critical pedagogy. Regarding borrowed theories, the one with the greatest concentration was the critical theory from Paulo Freire, who supports an educational approach centered on developing conscious critical thinking in students, a type of thinking that permits questioning beliefs and social practices that generate oppression.^26^ Thus, through this and the use of praxis, emancipation of those who are oppressed is achieved, given that the work with both will allow subjects to be critical about social reality.^27^ This theory is sustained by culture and the recognition of cultural diversity, understood as “differences in skin color, race, ethnicity, nationality, socioeconomic status, level of education, employment status, and religion”.^20^ Nevertheless, in the articles reviewed, this concept was addressed in nursing care and the theory by Freire is focused on education. In the nursing practice, it is expected for this view to support the transformation of teaching the profession,^28^ in the sense that it considers the professor as a companion and the student is assumed as an agent of social change, which in the educational interactions achieve the interpretation, transformation, and intervention of the problems and needs of the community.^29^

Eco-social model. At interdisciplinary model level, the eco-social model proposed by Krieger^30^ was the most referenced in the studies reviewed. This proposes a perspective centered on inequalities at health level, which are explained from a combination of social and biological factors.^16^ Knowing the influence of these factors permits not being restricted in the scope, addressing health characteristics, like space and time, multilevel interactions, and personal particularities.^26^ At conceptual level, this model was worked from the culture and encounters and cultural interaction, points retaken also in the disciplinary model of cultural competency.

Educational act. Overall, the studies indicated different points related with the educational act, formed by the curriculum, didactics, and evaluation.^31^ The findings concerning these are described in the following. In relation with the curriculum, inclusion was noted of components from cultural competency at theoretical level^32^ in the planning and programming of assignments.^33^ Regarding what is taught, real curriculum, the students experience the discussion around attributes required to be culturally competent and the different components of the term of cultural competency.^24^ Curricular pertinence is seen around globalization and changes in the population due to migration,^34^ where study plans require the inclusion of the demands of the context.^16^ The didactics suggested vary depending on the learning objectives, for example, Long^35^ emphasizes the need to use debates and essays that permit students to reflect upon an experience of intercultural encounter. The short-term and long-term cultural immersion is highlighted in various studies upon favoring the theory and practice relation in the interaction with people.^13^^,^^36^ Also indicated, are web tools, simulation, ^37^ and cases constructed by the community from social research, by providing forms of cultural interaction without ethical risks.^38^ The review also found that the evaluation of cultural competency is a highly relevant point, given that in formation institutions of nursing professionals, it permits recognizing the level of understanding and comprehension of other cultures.^39^^,^^40^ It is evident that there is still a need to consider that the topic requires being worked transversally and professors need specific preparation in theories and models to improve their pedagogical practices.^41^ There is marked interest in knowing the level of the acquired competences, where the validation of instruments was central; nonetheless, this is carried out partially as only certain components of cultural competency were addressed. The research findings reveal that when a person is from a culture other than the context where they are trained, they feel more confident in caring for diverse people.^34^

Gaps and recommendations on intercultural conceptual and theoretical models. As principal gaps, it was found that in the training practices conducted in clinical scenarios, a theoretical-practical gap persists when revealing difficulty in applying the models and theories.^42^^,^^43^ Likewise, a challenge is posed in the education of future nursing professionals when not having the types of evaluations that identify in the students if they have acquired the skills and knowledge proposed by the models and theories in relation with the development of cultural competency.^44^^,^^45^ Moreover, it is shown that research is needed in the educational setting with respect to how the teaching process contributes to the student’s training in the development of cultural competency. Table 2 indicates the other gaps identified, such invite us to ponder how academia can develop a cultural competency model articulated with care to, thus, achieve curricular pertinence from the context; it should be clarified that not all the works reviewed indicated gaps.

**Table 2:** Research gaps in nursing education in intercultural contexts

Gap	Number	%
Application in the practice	11	25.57
Evaluation of the competency in the practice	8	18.60
Research on education	7	16.28
Development of cultural competency during training	5	11.62
Clarity of the concept	3	6.98
Negative effects in clinical care	3	6.98
Student motivation	2	4.65
Scientific resources	1	2.33
Centered on the development of the competency	1	2.33
Concepts not retaken in clinical teaching	1	2.33
Scarce knowledge about cultures	1	2.33

With regards to the recommendations (Table 3), among the works that formulated recommendations, most indicate the need to train professors in intercultural theories and models because these require knowledge to be able to teach to others.^16^ In addition, the need was identified to include topics of cultural competency in curricula,^46^ given that it is poorly addressed in universities or is treated as a tangential issue, which in many cases is not approached adequately or is made invisible by remaining in the discourse.^20^

**Table 3:** Recommendations for nursing education in intercultural contexts

Recommendations indicated	Number	%
Teacher training	7	22.58
Inclusion of topics of cultural competency	6	19.35
Evaluation of programs	5	16.13
Experiential didactics	4	12.90
Own models and theories	3	9.68
Learning in context	2	6.45
Include ethics and moral reasoning	2	6.45
Research	1	3.23
Institutional commitment	1	3.23

## Discussion

The findings allow unveiling different achievements evidenced in the research of the nursing models and theories applied in intercultural education. Thus, the trends show an approach that denotes interest in the formation. At disciplinary level, the Campinha-Bacote model predominated, which “is limited, to the extent that the health provider-subject of care power relationship is maintained, recognizing the importance of culture in the subject’s health, but without leading to dialogue”.^47^ The situation does not favor care with a more reciprocal view, when keeping in mind that populations are increasingly more heterogenous, where care implies a process of empathy, openness, sympathy, and generosity.^4^ Consequently, trust relationships are expected to be built between subject of care and caregiver that imply time, knowing antecedents, language, knowledge regarding health care, and compassionate willingness to comprehend such.^48^ So, compassion in the pedagogical act provides, to whom it prepares and who is prepared, sensitivity to the pain or suffering of another (diverse but which is equal as human being), upon understanding that the actions of the nurse caregiver lead to consequences that impact on the subject and that this not only involves epistemic sustenance, but also attitudes and emotions.^48^^,^^49^

With respect to the interdisciplinary theories identified, and which contribute to the field of nursing education from an intercultural approach, these are guided from the contributions by Paulo Freire,^28^ who calls for the emancipation of pedagogy from dominant the classes to pedagogy of freedom, where the oppressed has the conditions to reflect, so that it is discovered and conquered. From this point of view, Flórez-Ochoa^50^ expresses that pedagogy is a theoretical and practical possibility that makes it easier for individuals to free themselves from themselves and from the context through the development of their conscious activity, that is, a process of humanization the authors denominate substance of pedagogical action. The process of humanization, in the opinion of Mendoza-Carrasco^51^ implies recognizing the student as a human being who needs affection, endearment, and respect, which they denominate pedagogy of love and tenderness. This pedagogy integrates science and spirituality because it advocates for a connection between reason and feeling, thus, educating is an act of mutual love.^51^

Hence, it is expected for nursing teaching within an intercultural context to permit the development of awareness and critical thinking,^52^ given that education is not a neutral process, but a way of reflecting about reality and think of ways to transform it, supported on the construction of curricula from dialogue with other disciplines.^26^ However, the studies reviewed do not delve into this last point; thereafter, the need is highlighted to retake the category of *interculturality* from borrowed theories that contribute to broadening the horizon in the nursing practice, bearing in mind that it addresses the care of human beings and the understanding of their conceptions, but it is also nourished by other perspectives.^16^

It is expected that a transdisciplinary approach, from the identity as nurses, not only to explore the concept of otherness, because it has already been identified who the others are, but to be able to know them during the formative process, by exploring the knowledge of others that is explicit within the disciplinary subjects, allowing to find common ground, not only from objective care, in terms of the development of skills, but from a subjective one in the acquisition of soft skills that promote a more human relationship from the recognition of others and other things.^5^

In effect, education in intercultural contexts must be more aware of the multicultural reality, where shocks emerge in health care, therefore, formation guided by own and adapted models and theories that respond to particular care is required.^4^ Hence, having an ethical stance is an essential requirement, which starts from the reflection on the relationship that emerges during care, the behavior, and commitment that must be had with that other, arising from the autonomy that provides an epistemic sustenance to assume the responsibility implied by care.^5^ Furthermore, nursing education in intercultural contexts has focused on topics, like: “Global nursing challenges, health care systems, transcultural theories and models, intercultural communication, beliefs, and practices based on culture, culture-based healing and care”.^15^ Consequently, some programs have adopted a specific model or theory that guides the formation of future professionals, as in the case of Slovenia with the transcultural nursing model and cultural competency.^15^ However, it is necessary to recognize that one is not more important than another, there are different contributions made from social sciences, philosophy, and nursing, which provide foundations to adapt to changes in populations around the world.^26^

Different achievements were found in the studies regarding the curriculum, such as recognition of the importance of flexibility and the practice within it. Thus, Prosen^15^ suggests that, for intercultural nursing formation, establishment of a flexible curriculum is required that adapts to the specific needs of the region, guides the forms of teaching and learning, besides facilitating during practices for students to interact with others who are culturally different. This situation from the ecological perspective of cultural competency requires early articulation between theoretical teaching and formative care practice, which favors dialogue with another and the search for solutions.^53^ Where the formative practice, as a learning scenario for students, can generate the recognition of their knowledge and the learning of other knowledge regarding health care, from the cultural shock that sometimes arises upon knowing diverse cultures and different from one’s own.^13^ Thereby, being able to relate in the clinical setting with patients and recognizing the difficulties that are met due to inequity or inequality in the quality of care received by individuals from minority groups,^54^ motivates future professionals to wanting to know the culture of the other, initiating the process of becoming culturally competent through cultural desire.^55^ Thus, academic units can think of their own model of intercultural care that guides training, bearing in mind the experiences of nursing professionals in the practice. 

The literature reports that when programs have an epistemic from where of nursing theory, or borrowed, that guides intercultural formation in its students, the didactics used most so that these can internalize such are cultural contacts in either formative practices or cultural immersions.^13^ An achievement that leaves a path to explore in academic units for each of them to analyze the time it takes the student and professor to develop this skill, considering the social, economic, and political context of each country. Curricula are required with specific assignments of intercultural theories and models, besides the use of didactics that facilitate their learning.^56^^,^^57^ Hence, inclusion of topics in the curriculum is not sufficient, given the difficulty to carry out culturally competent care in the institutions where students conduct the practice. Shoghi *et al*.,^42^ consider that there is lack of dialogue between the formation institutions and the context, underscoring the importance of understanding that: “the gap between theory and practice is a constant nursing problem experienced by advanced students and newly qualified professionals (…), which is summarized as the gap between the theories the professionals claim underlie their practice and the implicit theories, of which they may not be aware, integrated in their practice”.^42^

It is worth highlighting that, in the practice scenarios, this theory and practice dichotomy generates difficulties in the relations established with the subjects of care due to the lack of knowledge about the other and self that does not allow comprehension.^34^ In this regard, Flores *et al*.,^58^ reiterate that, in the university, from the teaching of health professionals, it becomes necessary to strengthen the different communication skills and openness attitudes necessary to provide intercultural care; thereby focusing on ways that allow knowing other visions of care, which highlight the importance of the context in health and the relevance both have in the assessment of the subject of nursing care.^13^ This leads to evaluating the acquisition of knowledge and skills, framed in the understanding of whether professionals are able to facilitate the relationship of care through understanding.^39^ Nevertheless, it is important to recognize in the cultural competency evaluation processes the need to transcend the doing and knowing the being, that is, consider that the professor’s and student’s experience caring for culturally diverse people is a challenge because in the care relationship tensions are experienced among linguistic barriers, knowledge, and ethical responsibilities.^5^

The methodologies used in the studies were principally qualitative, which shows the comprehensive approach that has been carried out; it is necessary to continue with this trend because it supports the study of interculturality by allowing to delve into the contextual and social depth of the theme^59^ when bearing in mind that care is based on relations. In addition, these methodologies permit knowing the patient’s conditioning factors through contextualization in their culture, which leads to an understanding of the perspective of what is health and disease, in order to improve the caregiver-subject of care relations.^4^

Regarding the limitations found, these were subdivided into the application and pedagogy. Thus, it is indicated that there is a marked difficulty in the application,^60^ students in care practice scenarios show lack of knowledge with respect to cultural competency, which leads to increased inequity of care.^15^ In pedagogy, scant teacher training is shown, given that teaching in nursing models and theories in intercultural contexts requires having an epistemic from where, which enables not only clarity of concepts but also lets the professor be a training model.^20^^,^^47^ Given the foregoing, processes of acculturation of minorities through adaptation to the environment are added in educational contexts ^61^ and cultural uncertainty in the care area, situations overcome with the inclusion of cultural competency during the formation.^13^ From Noddings’ point of view,^62^ to educate in cultural competency, it is necessary to recognize that caring is evidenced in the relations established with professors, students, and colleagues, for which moral education is favored in the pedagogical act, to the extent that, from the interactions with the professors, students receive respect and are listened to, establishing a dialogue that enables their developing skills and attitudes to maintain care relations with others. This implies the student’s active attitude in learning from dialogue-action.^63^ Thus, the importance is highlighted of including in the curriculum a more human perspective that opens the way to the ethical vision of care that permits moral reasoning, where the relationships that emerge in this interaction are prioritized to understand who others are, to recognize them and recognize oneself in interaction with diverse cultures.^24^

With respect to the gaps found, there is low student motivation to access the practice, which demands the commitment of accompaniment by educational institutions,^15^ not only with regards to the inclusion of subjects and teacher training, but that the choice of cultural immersions is facilitated or included within curricula, given that student motivation for these experiences is hindered by administrative difficulties and graduation delays.^13^ In addition to underscoring the importance of conducting research on intercultural contexts in the educational setting, with a low density of production in this area compared with the care area.^64^ Likewise, the gaps demonstrate that there is still a way to go at conceptualization level of models and theories because ambiguity is found in the terms that compose them, as in the case of cultural competency, where some author emphasize on competency and others on culture.^20^^,^^65^ This makes it necessary to propose own models and theories that recognize the diversity experienced, its causes, and the particularities of the context.^42^ This is so specially when the theories and models found have been addressed mainly in the United States, where diversity is related with migration, different from Latin American countries in which multiethnicity and pluriculturality are characteristic of the region; bearing in mind that culturally competent care is expected to enable more effective care, which improves health by diminishing inequity,^20^ facilitating the caregiver-subject of care interaction,^24^ which impacts upon the community.^34^ Thinking of formation that includes interculturality from the importance of having an ethical stance in care, where ethics provides epistemic and ontological support to nursing professionals that allow them to recognize their responsibilities and duties with another who is diverse and, consequently, broadens the vision of health care toward a more holistic vision of the human being.^(66, 5)^ The aforementioned invites us to think about theories and models from our own context, so they can be applied, more so when scant research is available regarding the practical utility of the models and theories established.^4^^,^^53^

Finally, as a recommendation, it seems important to rethink the internal and external pertinence of the curricula at the macro context level of the training of nursing professionals from interculturality, a point of origin would be the training of professors regarding cultural competency, exploring the knowledge of students and research in territories, to generate social impact in and out of the classroom. In turn, it is convenient for higher education institutions to meet students’ needs in relation to their interests of intercultural practice scenarios that contribute to graduation profiles that respond to the country’s reality. 

## Conclusion

Upon analyzing the academic production, it was found that ethical action by professionals is fundamental for the nursing task, highlighting that there is an ethical view of care by dimensioning that such cannot be understood as homogeneous, rather that concern exists in educating for particular care. However, coherence is required between theory and practice in education to close the existing gap in the application of models and theories, considering that these are based on caregiver-subject of care relations, but that clarity is needed on how to measure the acquisition of aptitudes and attitudes for care. The journey through the literature found permitted visualizing the trends, where these demonstrate that although a wide range of studies was found, there is still no application clarity, this delimited in that nursing programs still have no consensus on what theories and models to use to apply them. The overall invitation would be framed in rethinking these models and theories in context to, thus, bring them to dialogue with borrowed theories, opening the vision to contributions from other disciplines.

In this sense, professors are again the center, given that as those in charge of formation, they must be valid interlocutors, not only solo from their conceptual knowledge but from their work, so that students can see their ethical actions and that the relations that emerge in the pedagogical act show openness to others through dialogue and respect.

## References

[1] Berchid-Martínez F, Herrero-Hahn R, Hueso-Montoro C. (2017). Producción científica en enfermería transcultural en el periodo 2007-2014. Cult. Cuid..

[2] UNESCO (2015). Replantear la educación: ¿Hacia un bien común mundial? - Biblioteca Digital de la UNESCO.

[3] Fornet-Betancourt R. (2006). La interculturalidad a prueba.

[4] Rubio Martín S, Rubio Martín S (2020). Diversidad cultural en salud, competencia de la Enfermería transcultural. Enferm. Cardiol..

[5] Valdéz-Fernández AL (2020). Sentidos sobre la bioética que emergen en las prácticas formativas de enfermería en un contexto intercultural.

[6] Beuchot M. (2005). Interculturalidad y derechos humanos.

[7] Campos Navarro R. (2010). La interculturalidad, la medicina tradicional y los trabajadores de la salud. Medicina Intercultural.

[8] Bueno Robles LS. (2011). Aspectos Ontológicos y Epistemológicos de las Visiones de Enfermería Inmersas en el Quehacer Profesional. Cienc. Enferm..

[9] Barret E. (1998). Theory: of or for Nursing?. Nurs. Sci. Q..

[10] Chrizostimo MM, Brandão AAP (2015). La formación profesional del enfermero: ʻestado del arteʼ. Enferm. Glob..

[11] Rojas-Reyes J, Rivera Álvarez LN, Medina Moya J (2019). Los currículos en enfermería y el desarrollo de las Competencias interpersonales: el caso de Colombia. Índex Enferm..

[12] Guevara Patiño R. (2016). El estado del arte en la investigación: ¿análisis de los conocimientos acumulados o indagación por nuevos sentidos?. Folios.

[13] Ferranto MLG. (2015). A Qualitative Study of Baccalaureate Nursing Students Following an Eight-day International Cultural Experience in Tanzania: Cultural Humility as an Outcome. Procedia Soc. Behav. Sci..

[14] Cerezo PG, Galceran MS, Soriano MG, Camps LM, Moral JL (2014). Design and Evaluation of an Educational Course in Cultural Competence for Nursing. Procedia - Soc. Behav. Sci..

[15] Prosen M. (2015). Introducing Transcultural Nursing Education: Implementation of Transcultural Nursing in the Postgraduate Nursing Curriculum. Procedia Soc. Behav. Sci..

[16] Drevdahl DJ. (2018). Culture Shifts: From Cultural to Structural Theorizing in Nursing. Nurs. Res..

[17] Williamson M, Harrison L (2010). Providing culturally appropriate care: A literature review. Int. J. Nurs. Stud..

[18] Cai D, Kunaviktikul W, Klunklin A, Sripusanapan A, Avant PK (2017). Identifying the essential components of cultural competence in a Chinese nursing context: a qualitative study. Nurs. Health Sci..

[19] Cai DY. (2016). A concept analysis of cultural competence. Int. J. Nurs. Sci..

[20] Sharifi N, Adib-Hajbaghery M, Najafi M (2019). Cultural competence in nursing: A concept analysis. Int. J. Nurs. Stud..

[21] Ahn JW. (2017). Structural Equation Modeling of Cultural Competence of Nurses Caring for Foreign Patients. Asian Nurs Res (Korean Soc Nurs Sci).

[22] Bauer K, Bai Y (2015). Innovative Educational Activities Using a Model to Improve Cultural Competency among Graduate Students. Procedia Soc. Behav. Sci..

[23] Purnell L. (2002). The Purnell Model for Cultural Competence. J. Transcult. Nurs..

[24] Henderson S, Horne M, Hills R, Kendall E (2018). Cultural competence in healthcare in the community: A concept analysis. Heal Soc Care Community..

[25] Xu ES, Wang R, Su YH, Wu YY, Liu X, Duan GX (2016). A preliminary multicultural nursing competence instrument for assessing undergraduate student nurses. Int J Nurs Sci..

[26] Andrews M, Backstrand JR, Boyle JS, Campinha-Bacote J, Davidhizar RE, Doutrich D (2010). Chapter 3: Theoretical Basis for Transcultural Care. J Transcult Nurs..

[27] Moreno Mojica CM, Barragán Becerra JA (2019). Prácticas pedagógicas y procesos de aprendizaje: configuración e institucionalización en la disciplina de enfermería. Ánfora..

[28] Freire P. (2005). Pedagogía del Oprimido.

[29] Mojica Moreno MC, Becerra Barragán AJ (2019). Pedagogical Practices and Learning Processes: Configuration and Institutionalization in the Discipline of Nursing. Ánfora..

[30] Krieger N. (2016). Living and Dying at the Crossroads: Racism, Embodiment, and Why Theory Is Essential for a Public Health of Consequence. Am J Public Health..

[31] Bohórquez FF, Corchuelo MH (2005). Currículo y pedagogía en perspectiva: un diálogo académico. Rev. ieRed..

[32] Kardong-Edgren S, Cason CL, Brennan AMW, Reifsnider E, Hummel F, Mancini M (2010). Cultural competency: Graduating BSN nursing students. Nurs. Educ. Perspect..

[33] Malagón L. (2005). El Currículo: Una Reflexión Crítica. Sophia..

[34] Vázquez-Sánchez MÁ, Casals C, Casals-Vázquez A, García-Barrios S, Fernández-de-Canete F, Sánchez-Ojeda MA (2021). Cultural adaptation and validation of the Transcultural Self-Efficacy Tool for use with undergraduate nursing students in Spain. Nurse Educ. Today..

[35] Long TB. (2012). Overview of teaching strategies for cultural competence in nursing students. J. Cult. Divers..

[36] Flavell H, Thackrah R, Hoffman J (2013). Developing Indigenous Australian cultural competence: A model for implementing Indigenous content into curricula. J. Teach. Learn. Grad. Employability..

[37] Marja SL, Suvi A. (2021). Cultural competence learning of the health care students using simulation pedagogy: An integrative review. Nurse Educ. Pract..

[38] Mathew L, Brewer BB, Crist JD, Poedel RJ (2017). Designing a Virtual Simulation Case for Cultural Competence Using a Community-Based Participatory Research Approach: A Puerto Rican Case. Nurse Educ..

[39] Papadopoulos I, Taylor G, Ali S, Al E (2017). Exploring Nurses’ Meaning and Experiences of Compassion: An International Online Survey Involving 15 Countries. J. Transcult. Nurs..

[40] García Navarro EB. (2015). Competencia cultural en salud: conocimientos, prácticas Culturales, y actitudes ante los cuidados.

[41] Gallagher RW, Polanin JR (2015). A meta-analysis of educational interventions designed to enhance cultural competence in professional nurses and nursing students. Nurse Educ. Today..

[42] Shoghi M, Sajadi M, Oskuie F, Dehnad A, Borimnejad L (2019). Strategies for bridging the theory-practice gap from the perspective of nursing experts. Heliyon..

[43] Unver V, Uslu Y, Kocatepe V, Kuguoglu S (2019). Evaluation of cultural sensitivity in healthcare service among nursing students. Eur. J. Educ. Res..

[44] Lapham KB. (2019). The Impact of an Evidence-Based Cultural Competence Workshop for University Student Health Centers’ Licensed Personnel.

[45] Tosun B, Yava A, Dirgar E, Şahin EB, Yılmaz EB, Papp K (2021). Addressing the effects of transcultural nursing education on nursing students’ cultural competence: A systematic review. Nurse Educ. Pract..

[46] Jesse DE, Kirkpatrick MK (2013). Catching the Spirit of Cultural Care: A Midwifery Exemplar. J Midwifery Women’ Health..

[47] Valdéz-Fernández AL. (2019). Interculturality: A position on training of nursing professionals. Cult. Cuid..

[48] Reina Leal LM, López Díaz L (2020). Entrelazando la compasión y la competencia cultural en hospitalización: Una revisión de experiencias. Cult. Cuid..

[49] Crawford P, Brown B, Kvangarsnes M, Gilbert P (2014). The design of compassionate care. J. Clin. Nurs..

[50] Flórez Ochoa R. (2013). El optimismo de la pedagogía. Rev. Educ. Pedagog..

[51] Mendoza Carrasco MV (2019). La pedagogía del amor y de la ternura, en las aulas hospitalarias del Perú. Educación.

[52] Epp S, Reekie M, Denison J, de Bosch Kemper N, Willson M, Marck P (2021). Radical transformation: Embracing constructivism and pedagogy for an innovative nursing curriculum. J. Prof. Nurs..

[53] Smith CS, Morris M, Langois-Winkle F, Hill W, Francovich C (2010). A pilot study using Cultural Consensus Analysis to measure Systems-Based Practice performance. Int. J. Med. Educ..

[54] Paynter Deal K. (2014). Breaking barriers in provider-patient relationships: an analysis of perceived intercultural communication competence among nursing students.

[55] Rivera-Goba M, Campinha-Bacote J (2008). Making a Connection: The Use of Storytelling as a Strategy to Enhance Faculty’s Success with Latina Nursing Students. Hisp. Health Care Int..

[56] Brommelsiek M, Peterson JA, Amelung SK (2018). Improving cultural competency: A patient-centered approach to interprofessional education and practice in a veteran’s healthcare facility. Int. J. High. Educ..

[57] Sánchez Sanabria M, Rondón Contreras BJ (2013). La diversidad cultural en los procesos de formación académica de enfermería requiere el manejo de la ética pedagógica, la corresponsabilidad y un pensamiento mediador. Enferm. Glob..

[58] Flores G, Laws MB, Mayo SJ, Zuckerman B, Abreu M, Medina L (2003). Errors in medical interpretation and their potential clinical consequences in pediatric encounters. Pediatrics..

[59] Cárcamo Ortiz MP (2009). Una propuesta de potenciación de los programas de salud intercultural, desde la perspectiva de la enfermería transcultural.

[60] Adamshick P, August-Brady M (2012). Reclaiming the Essence of Nursing: The Meaning of an Immersion Experience in Honduras for RN to Bachelor of Science Students. J. Prof. Nurs..

[61] Vallee KE. (2018). Supporting Indigenous students: A critical analysis of the sociocultural context of nursing education.

[62] Noddings N. (2005). The challenge to care in schools.

[63] Valdéz Fernández AL (2020). Sentidos de la formación bioética de enfermeros en un contexto intercultural. Cult. Cuid..

[64] Rowan MS, Rukholm E, Bourque-Bearskin L, Baker C, Voyageur E, Robitaille A (2013). Cultural competence and cultural safety in Canadian Schools of Nursing: A mixed methods study. Int. J. Nurs. Educ. Scholarsh..

[65] Burns S. (2020). California State University (CSU) System nursing faculty: Are you culturally competent to teach in a multicultural state?. J. Prof. Nurs..

[66] De Moura C, Lucia M (2005). Enseñanza de Competencia y para Competencia en Enfermería. Enferm. Glob..

